# Increasing transforming growth factor-beta concentrations with age decrease apelin in the rat rotator cuff

**DOI:** 10.1186/s13018-021-02675-0

**Published:** 2021-08-31

**Authors:** Ryo Tazawa, Kentaro Uchida, Tomonori Kenmoku, Mitsufumi Nakawaki, Kyoko Muneshige, Daisuke Ishii, Gen Inoue, Masashi Takaso

**Affiliations:** Department of Orthopedic Surgery, Kitasato University School of Medicine, 1-15-1 Kitasato, Minami-ku, Sagamihara, Kanagawa 252-0374 Japan

**Keywords:** Rotator cuff degeneration, Transforming growth factor-beta, Apelin

## Abstract

**Background:**

The rotator cuff undergoes natural degeneration with age, leading to age-related rotator cuff tear; however, the precise mechanism remains unclear. Transforming growth factor-beta (TGF-β) concentrations rise with age and TGF-β contributes to the pathophysiology of skeletal muscle. TGF-β has also been shown to suppress expression of the myokine, apelin, in skin fibroblasts. We hypothesized that TGF-β expression in the rotator cuff changes with age and regulates apelin expression, thereby contributing to rotator cuff degeneration.

**Methods:**

We used quantitative reverse-transcription polymerase chain reaction (Q-RT-PCR) to measure the expression of apelin and tendon-related genes (*Tnmd*, *Col1a1*, and *Col3a1*) in the rotator cuff of young (12 weeks), adult (24 weeks), and old (48 weeks) rats. Using Q-RT-PCR and enzyme-linked immunosorbent assay, we also measured *Tgfb* mRNA and TGF-β protein levels, respectively. Furthermore, we used Q-RT-PCR to measure apelin mRNA levels in rotator cuff-derived cells after treatment with 0 (control) and 10 ng/mL recombinant TGF-β.

**Results:**

Apelin mRNA levels were significantly lower in old compared to young and adult rats. Similarly, tendon-related genes, *Tnmd*, *Col1a1*, and *Col3a1*, were significantly lower in adult and old rats than young rats. In contrast, *Tgfb* mRNA and TGF-β protein were significantly higher in old compared to young rats. Stimulation with exogenous TGF-β significantly decreased *Apelin* mRNA expression compared to control.

**Conclusions:**

TGF-β regulates apelin expression in the rotator cuff and may play a key role in the degenerative pathology of the rotator cuff with age.

## Background

The rotator cuff consists of four muscle/tendon units, the supraspinatus, infraspinatus, subscapularis, and teres minor, originate at the scapula and insert into the proximal humerus [1]. The supraspinatus is responsible for initiating abduction of the glenohumeral joint (GHJ) [2], the infraspinatus and teres minor for external rotation, and the subscapularis for internal rotation of the GHJ [2, 3]. They work in synergy to stabilize the GHJ [3]. Rotator cuff tears (RCT) are often associated with severe shoulder pain and shoulder dysfunction that lead to activity of daily living disability [4–6]. Evidence suggests [7, 8] that a combination of extrinsic factors such as greater subacromial loading [9] and muscle imbalance, and intrinsic factors such as age-related degeneration [10, 11] contribute to the development of RCT. Prior studies indicate that RCTs can occur naturally with age [10–12]. Thus, the incidence of RCT rises with age, with approximately 30% of cases aged ≥ 60 years [13]. However, it remains unclear how age-related rotator cuff degeneration arises.

Transforming growth factor-beta (TGF-β) is a multifunctional cytokine with roles in cell growth, differentiation, and apoptosis [14]. Additionally, TGF-β contributes to the pathogenesis of fibrosis in the lungs, liver, kidney, heart, and skeletal muscle, as well as most other organs, and is thought to play a role in a number of diseases and syndromes [15]. Gene expression of TGF-β1 in skeletal muscle is reportedly elevated in older compared to younger adults [16]. However, whether TGF-β increases with age in the rotator cuff remains unknown.

Production of apelin, a natural ligand of the angiotensin-1-like receptor (also known as the apelin receptor: APJ) [17], is reduced with age. Apelin reverses age-associated sarcopenia [18] and the interaction between apelin and APJ has therapeutic effects in renal fibrosis, cardiac fibrosis, and pulmonary fibrosis [19]. Interestingly, TGF-β stimulation decreases apelin production in fibroblasts [20]. We thus hypothesized that TGF-β expression in the rotator cuff changes with age and regulates apelin expression, thereby contributing to degeneration of the rotator cuff.

We examined age-related changes in TGF-β and apelin expression and the relationship between TGF-β and apelin in the rat rotator cuff.

## Methods

### Animals

Previous studies have reported that the supraspinatus tendon in the rat shoulder is structurally similar to the human coracoacromial arch, and that a rat rotator cuff injury model mimics human disease syndromes and provides reproducible results [21, 22]. Therefore, we used a rat rotator cuff in this study. The study protocol was approved by Kitasato institutional Animal Care Committee (Permission number: 2020-091). The study was carried out in compliance with the ARRIVE guidelines for the reporting of animal experiments. All methods were carried out in accordance with the guidelines for the proper conduct of animal experiments by the Science Council of Japan. Male Wistar rats (Charles River Laboratories Japan, Inc., Yokohama, Japan) were provided a commercial pelleted diet (CRF-1, Oriental Yeast Industry, Tokyo).

### Quantitative reverse-transcription polymerase chain reaction (Q-RT-PCR) analysis

**Fig. 1:**
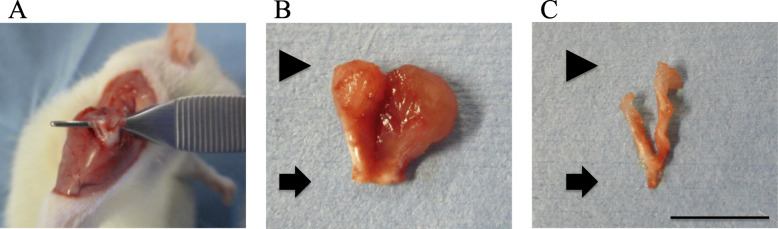
Harvesting of the supraspinatus and infraspinatus tendons from rats. The supraspinatus and infraspinatus muscles and tendons were harvested from rats (**A**, **B**). The muscle component was stripped off, leaving only the tendons for use in the experiments (**C**). Arrows indicate the glenoid side; arrowheads indicate the humeral side of the tendon. Scale bar indicates 10 mm

We examined age-related changes in gene expression by first dividing the animals into three groups based on age: young (12 weeks), adult (24 weeks), and old (48 weeks) (*n* = 9 each). Right supraspinatus and infraspinatus tendons were extracted from each rat under isoflurane anesthesia (Fig. 1). Total RNA from rotator cuff samples was extracted using TRIzol (Invitrogen, Carlsbad, CA, USA) according to the manufacturer’s instructions. First-strand cDNA synthesis was conducted using SuperScript III RT (Invitrogen) with the extracted RNA as a template, 2 μL cDNA, a specific primer set (0.2 μM final concentration), and 12.5 μL SYBR Premix Ex Taq (Takara, Shiga, Japan) in a total volume of 25 μL. Primers for *Tgfb*, *Apelin*, and tendon-related genes (*Tnmd*, *Col1a1*, and *Col3a1*), the sequences of which are provided in Table 1, were designed using Primer Blast and produced by Hokkaido System Science Co., Ltd. (Sapporo, Japan). Primer-amplified products were checked for specificity using melt curve analysis. Q-RT-PCR was performed using a CFX-96 Real-Time PCR Detection System (Bio-Rad, Hercules, CA, USA) with the following parameters: initial denaturation at 95 °C for 1 min, and 40 cycles at 95 °C for 5 s and 60 °C for 30 s. Levels of *Tgfb*, *Apelin*, *Tnmd*, *Col1a1*, and *Col3a1* mRNA in the rotator cuff were ascertained by normalizing to levels of glyceraldehyde-3-phosphate dehydrogenase using the delta-delta Ct method. Relative expression was determined based on the average of all control samples (rotator cuff samples taken from young rats).

**Table 1:** Sequences of the primers used in this study

Primer	Sequence (5′-3′)	Product size (bp)
*Tgfb*-F	GACCGCAACAACGCAATCTA	110
*Tgfb*-R	GACAGCAATGGGGGTTCTGG	
*Apelin*-F	CTTGACTGCCGTGTGTGGA	72
*Apelin*-R	CGCATGTTGCCTTCTTCTAGC	
*Tnmd*-F	AACAAATCGTAGCACGGGAG	102
*Tnmd*-R	AGTCGGCTAACAGATGCCAG	
*Col1a1*-F	GTCCCTAATGGTGAGACGTGG	122
*Col1a1*-R	CGTTTTTGGGGGTTGGGACA	
*Col3a1*-F	ACACCTGCTCCTGTCATTCC	99
*Col3a1*-R	AAGACCAGGGTCGCCATTTC	
*Gapdh*-F	TGCCACTCAGAAGACTGTGG	129
*Gapdh*-R	TTCAGCTCTGGGATGACCTT	

### Enzyme-linked immunosorbent assay (ELISA) for TGF-β

We examined age-related changes in TGF-β protein levels in tissue taken from young, adult, and old rats (*n* = 9 each), grouped as described above, using ELISA. Left supraspinatus and infraspinatus tendons were extracted from rats as indicated above and homogenized in radioimmune precipitation buffer (Wako Pure Chemical Co., Inc., Osaka, Japan) containing a protease inhibitor cocktail (Roche, Madison WI, USA). Total protein was determined using the bicinchoninic acid assay (Thermo Fisher Scientific, Rockford IL, USA) and TGF-β protein using a TGF-β ELISA kit (R&D Systems, Minneapolis, MN, USA).

### Effect of TGF-β on apelin expression in rotator cuff-derived cells

Bilateral supraspinatus and infraspinatus tendons were harvested from 12-week-old Wistar rats as indicated above and digested with type I collagenase overnight at 37 °C to extract rotator cuff-derived cells. After 1 week of culturing in α-MEM supplemented with 10% fetal bovine serum at 37 °C in a 5% CO_2_ incubator, the cells reached confluence. Recombinant TGF-β (R&D Systems) was diluted with α-MEM supplemented with 1% fetal bovine serum. The cells were then subjected to simulation with recombinant TGF-β (0 and 10 ng/mL) for 6 and 24 h. Total RNA was extracted from treated (10 ng/mL TGF-β) and control (0 ng/mL TGF-β) cells, and *Apelin* expression was determined using Q-RT-PCR.

### Statistical analysis

Findings from rats in the three age groups were compared using Fisher’s LSD test, and those between treated and control rotator cuff-derived cells were compared using Student’s *t* test. Statistical analysis was conducted using SPSS (Version 19.0; SPSS, Inc., Chicago, IL, USA), and *p* < 0.05 indicated statistical significance.

## Results

### Expression of apelin and tendon-related genes in the rotator cuff

**Fig. 2:**
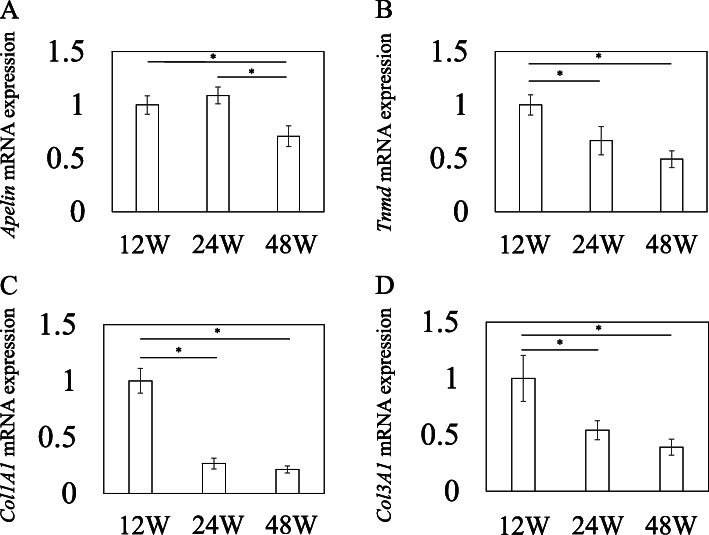
Quantitative reverse-transcription polymerase chain reaction analysis of apelin and tendon-related gene expression in the rotator cuff. Relative gene expression of *Apelin* (**A**) and tendon-related genes *Tnmd* (**B**), *Col1a1* (**C**), and *Col3a1* (**D**) in rotator cuff cells extracted from young (12 weeks), adult (24 weeks), and old (48 weeks) rats. Data represent mean ± SE (*n* = 9). * *p* < 0.05, among three groups

Changes in *Apelin* and tendon-related genes, *Tnmd*, *Col1a1*, and *Col3a1*, in the rotator cuff with age were examined using Q-RT-PCR. *Apelin* mRNA in old rats was significantly lower than that in young and adult rats (*p* = 0.027 and *p* = 0.005, respectively; Fig. 2A). In addition, levels of tendon-related genes, *Tnmd*, *Col1a1*, and *Col3a1*, were significantly lower in adult and old rats than in young rats (*Tnmd*, *p* = 0.033 and *p* = 0.002; *Col1a1*, *p* < 0.001 and *p* < 0.001; *Col3a1*, *p* = 0.024 and *p* = 0.004, respectively; Fig. 2B–D).

### *Tgfb* expression and TGF-β protein concentration in the rotator cuff

**Fig. 3:**
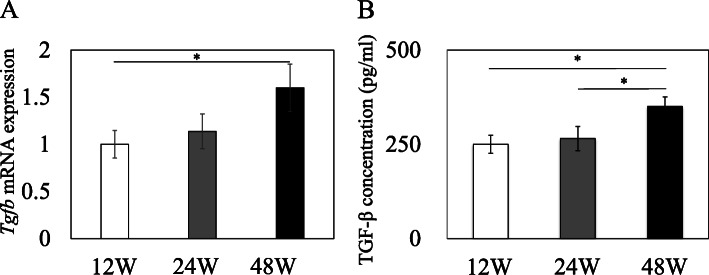
TGF-β gene expression and TGF-β protein concentration in the rotator cuff. (**A**) Relative *Tgfb* gene expression in rotator cuff cells extracted from young (12 weeks), adult (24 weeks), and old (48 weeks) rats. (**B**) TGF-β protein concentration in rotator cuff cells from young (12 weeks), adult (24 weeks), and old (48 weeks) rats. Data indicate mean ± SE (n = 9). * *p* < 0.05, among three groups

We performed Q-RT-PCR and ELISA to determine whether *Tgfb* mRNA and TGF-β protein levels rise in the rotator cuff with age. *Tgfb* mRNA expression in old rats was significantly higher than that in young rats (*p* = 0.043; Fig. 3A). Similarly, TGF-β protein was significantly higher in old than young and adult rats (*p* = 0.016 and *p* = 0.038, respectively; Fig. 3B).

### Effect of TGF-β on apelin expression in rotator cuff-derived cells

**Fig. 4:**
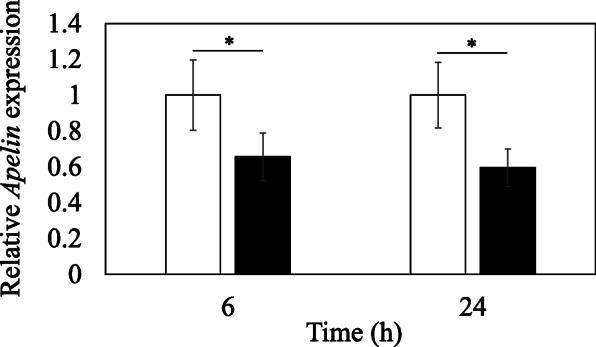
Effect of TGF-β on apelin mRNA expression. Effect of TGF-β on *Apelin* gene expression in cultured rotator cuff-derived cells. Rotator cuff-derived cells were stimulated with 0 (control) and 10 ng/mL recombinant TGF-β. Gene expression was compared between cells that received TGF-β stimulation for 6 and 24 h and the control group (0 ng/mL TGF-β). Data indicate mean ± SE (*n* = 9). * *p* < 0.05, compared to control

We stimulated rotator cuff-derived cells with TGF-β in vitro to determine the relationship between TGF-β and apelin. According to Q-RT-PCR analysis, stimulation with exogenous TGF-β for 6 and 24 h significantly reduced *Apelin* mRNA expression relative to control (*p* = 0.001 and *p* = 0.007, respectively; Fig. 4).

## Discussion

We investigated age-related changes in TGF-β and apelin expression with the aim of determining the relationship between these factors in the rat rotator cuff. Levels of *Tgfb* gene and protein rose, while those of *Apelin* and tendon-related genes declined with age in the rat rotator cuff. Further, exposing rotator cuff-derived cells to exogenous TGF-β led to a significant decline in *Apelin* levels.

A previous study showed that TGF-β1 concentrations in muscle cells increase during the normal aging process and in the transition to a more-fibrotic phenotype [23]. However, elevated TGF-β1 concentrations inhibit satellite cell activation and impair myocyte differentiation [24]. Meanwhile, SB431542, a TGF-β small molecule inhibitor, has been shown to reverse rotator cuff muscle fibrosis and fatty infiltration by inducing apoptosis of fibro/adipogenic progenitors [25]. The extracellular matrix of the supraspinatus tendon is primarily made up of type I and type III collagen. Several studies have described the pathological changes in rotator cuff degeneration, including the thinning and disorientation of collagen fibers, and diminished cellularity, vascularity, and fibrocartilage mass at the cuff insertion site [26, 27]. Tenomodulin (Tnmd) is expressed in tendon progenitor cells throughout lineage differentiation and is needed for collagen fibril maturation. When Tnmd is missing from tendons, the caliber of collagen fibrils increases, indicating impaired maturation [28, 29]. Here, we showed that tendon-related gene expression decreased as TGF-β increased with age in the rat rotator cuff, suggesting a link between changes in TGF-β expression and degenerative changes in the rotator cuff with age.

Muscle contraction-mediated apelin production declines with age. Apelin reverses age-associated sarcopenia and apelin treatment increases muscle mass in aged wild-type and *Apln−/−* mice, a function that is linked to fiber hypertrophy [18]. Interestingly, stimulation with TGF-β reduces apelin production in skin fibroblasts [20]. A previous study reported that the apelin-APJ axis inhibits fibrosis in several organs and has a synergistic effect on attenuating renal fibrosis [30]. Here, we found that *Apelin* and tendon-related gene expression decreased with age in the rat rotator cuff and that stimulation of rotator cuff-derived cells with exogenous TGF-β significantly decreased *Apelin* gene expression. Together, these findings suggest that TGF-β regulates apelin expression in the rotator cuff and may thereby induce rotator cuff degeneration with age.

Several limitations in this study warrant mention. We did not directly demonstrate degeneration of the rotator cuff with age; rather, we inferred this based on the decrease in tendon-related gene expression. Hence, further experiments such as tensile testing of the rotator cuff should be conducted to explore differences in the biomechanical properties among age groups. Second, the origins of apelin production in the rotator cuff require further study. Third, our study was conducted in rats, which are small animals. Thus, our findings may not be directly applicable to humans. Further studies using large animals or humans are needed to determine the degenerative pathology of the rotator cuff with age.

## Conclusions

We showed that there is an age-related increase in TGF-β and decrease in apelin and tendon-related genes in the rat rotator cuff. TGF-β suppressed apelin expression in rotator cuff-derived cells, suggesting that it may play a key role in the degenerative pathology of the rotator cuff with age.

## Data Availability

The datasets supporting the conclusions of this article are included within the article. The raw data can be requested from the corresponding author.

## Abbreviations

APJ: Apelin receptor
ELISA: Enzyme-linked immunosorbent assay
GHJ: Glenohumeral joint
Q-RT-PCR: Quantitative reverse-transcription polymerase chain reaction
RCT: Rotator cuff tear
SE: Standard error
TGF-β: Transforming growth factor-beta
